# Deuterated Polyunsaturated Fatty Acids Reduce Oxidative Stress and Extend the Lifespan of *C. elegans*

**DOI:** 10.3389/fphys.2019.00641

**Published:** 2019-05-28

**Authors:** Caroline Beaudoin-Chabot, Lei Wang, Alexey V. Smarun, Dragoslav Vidović, Mikhail S. Shchepinov, Guillaume Thibault

**Affiliations:** ^1^School of Biological Sciences, Nanyang Technological University, Singapore, Singapore; ^2^Retrotope Inc., Los Altos, CA, United States; ^3^School of Chemistry, Monash University, Melbourne, VIC, Australia

**Keywords:** polyunsaturated fatty acid (PUFA), deuterated fatty acid, oxidative stress, lipid peroxidation, lifespan, *C. elegans*, essential fatty acids, linolenic acid

## Abstract

Chemically reinforced essential fatty acids (FAs) promise to fight numerous age-related diseases including Alzheimer’s, Friedreich’s ataxia and other neurological conditions. The reinforcement is achieved by substituting the atoms of hydrogen at the bis-allylic methylene of these essential FAs with the isotope deuterium. This substitution leads to a significantly slower oxidation due to the kinetic isotope effect, inhibiting membrane damage. The approach has the advantage of preventing the harmful accumulation of reactive oxygen species (ROS) by inhibiting the propagation of lipid peroxidation while antioxidants potentially neutralize beneficial oxidative species. Here, we developed a model system to mimic the human dietary requirement of omega-3 in *Caenorhabditis elegans* to study the role of deuterated polyunsaturated fatty acids (D-PUFAs). Deuterated trilinolenin [D-TG(54:9)] was sufficient to prevent the accumulation of lipid peroxides and to reduce the accumulation or ROS. Moreover, D-TG(54:9) significantly extended the lifespan of worms under normal and oxidative stress conditions. These findings demonstrate that D-PUFAs can be used as a food supplement to decelerate the aging process, resulting in extended lifespan.

## Introduction

Sensitive to oxidative damage, the brain consumes around 20% of oxygen despite making only 2% of body weight. The brain is rich in polyunsaturated fatty acids (PUFAs) and oxygen in the lipid bilayer is particularly high reaching millimolar levels (Subczynski and Hyde, 1983). This tissue requires large quantity of ATP to maintain intracellular ion homeostasis resulting in high oxygen uptake. Iron also accumulates in the brain becoming problematic in late-life by catalyzing free radical reactions (Zecca et al., 2004). As the brain produces more mitochondria-generated superoxide compared to skeletal muscle, neuron produces more reactive oxygen species (ROS) with low levels of endogenous antioxidants (Malinska et al., 2009). As a result, the brain spends a quarter of its energy to maintain and repair lipid membranes damaged from ROS (Brenna and Carlson, 2014). Several studies demonstrated that blocking the production of lipid peroxides can be beneficial to prevent the development of Alzheimer’s disease (AD), Parkinson’s disease, and Huntington’s disease (Huang et al., 1999; Lee et al., 2011; Reed, 2011; Gandhi et al., 2012; Shichiri, 2014; Deas et al., 2016).

Senescence is a major cause of age-related diseases, correlating with an accumulation of ROS (McHugh and Gil, 2018). Enhanced formation of ROS oxidizes lipids to generate peroxides and aldehydes. Unlike short-lived ROS, these lipid peroxidation (LPO) products can produce damage throughout the cell due to their non-radical nature. Thus, lipid peroxidation is also strongly linked to aging and the accumulation of LPO products has been observed in AD, Parkinson’s disease, stroke, rheumatic arthritis, and cancer (Davies and Guo, 2014; Shichiri, 2014). However, clinical studies on the efficiency of antioxidants for these age-associated diseases have been disappointing.

The rate liming step of PUFA autoxidation is an ROS-driven hydrogen abstraction off a bis-allylic (between double bounds) methylene group, which is followed quickly by a series of transformations, generating toxic end-products (Shchepinov, 2007). During the nineties, there was a prominent research interest in dietary antioxidants generating hope as potential agent to slow aging and to prevent the development of ROS-related diseases. Disappointing results emerged from the studies conducted in humans (Moller and Loft, 2002). Thus, it appeared that human cells generate a certain amount of beneficial free radicals, playing important roles in cellular functions (Halliwell, 2011; Sena and Chandel, 2012; Yan, 2014). A balance of antioxidants and ROS must be kept *in vivo*, and supplementation of dietary antioxidants might compromise this critical equilibrium (Sies et al., 2017). This may be due to several reasons, including (1) the near-saturating amount of antioxidants already present in living cells and the stochastic nature of the ROS-inflicted damage, (2) the importance of ROS in cell signaling and hormetic upregulation of protective mechanisms, (3) the pro-oxidant nature of some antioxidants such as vitamin E, and (4) the non-radical nature of PUFA peroxidation products, which can no longer be quenched with most antioxidants (Shchepinov, 2011).

**FIGURE 1:**
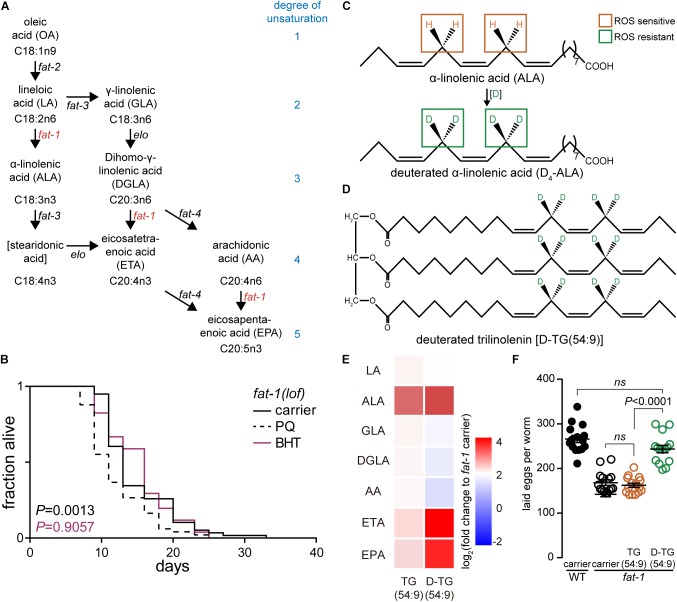
Deuterated trilinolenin rescues *fat-1* infertility phenotype. **(A)** Polyunsaturated fatty acid (PUFA) biosynthesis in *C. elegans*. Key enzymes *fat-1* and *fat-2* are highlighted in red. Adapted from Shmookler Reis et al. (2011). **(B)** Lifespan assay of *fat-1*(*lof*) subjected to carrier, 0.2 mM butylated hydroxytoluene (BHT), or 1 mM PQ. Log-rank test to carrier. **(C)** α-linolenic acid (ALA) is susceptible to oxidation attacks while deuterated ALA (D4-ALA) is protected. **(D)** Schematic representation of deuterated trilinolenin [D-TG(54:9)]. **(E)** Heat map of PUFAs extracted in *fat-1*(*lof*) supplemented with carrier, 0.48 mM trilinolenin [TG(54:9)] or 0.48 mM deuterated trilinolenin [D-TG(54:9)]. **(F)** Number of laid eggs per worm of WT and *fat-1*(*lof*) animals treated as in **(E)**.

Mitochondrial membranes are rich in cardiolipin, predominantly containing linoleic acid (LA) and α-linolenic acid (ALA) FAs (Paradies et al., 2002; Figure 1A). Thus, as PUFAs are essential nutrients in human, supplementing a diet with deuterated bis-allylic methylenes could represent the most promising approach to fight against ROS-initiated attacks leading to alleviate the influence of aging and age-associated diseases on human life. In *Saccharomyces cerevisiae*, deuterated PUFAs (D-PUFAs) have been shown to reduce oxidative stress (Hill et al., 2011) by protecting mitochondria against ROS (Andreyev et al., 2015). In addition, D-PUFA prevented lipid peroxidation in primary co-cultures of neurons and astrocytes (Angelova et al., 2015) and in Friedreich ataxia model system (Cotticelli et al., 2013). More recently, D-PUFAs significantly ameliorated performance in cognitive and memory tests using the AD mouse model *Aldh2^-/-^* (Elharram et al., 2017). This recent finding suggests that D-PUFAs might be sufficient to reduce the generation of AD-induced lipid peroxidation and to prevent the cognitive decline in AD. In human, a recent randomized clinical trial has been conducted, demonstrating the safety and potential protective effect against Friedreich’s ataxia (Zesiewicz et al., 2018). Friedreich’s ataxia is a neurodegenerative disease associated with an increase of oxidative stress (Ventura et al., 2009). More than a decade ago, supplementing the diet with D-PUFAs was predicted to delay aging (Shchepinov, 2007). However, there is still no study reporting the role of D-PUFAs in modulating lifespan.

In this study, we characterized the role of D-PUFAs against ROS in the multicellular model organism *C. elegans*. This organism was recently reported to be sensitive to deuterated lysine (5,5-D_2_-lysine) which strongly affected its development (Korneenko et al., 2017). To mimic the dietary requirement of omega-3 in human, we selected the omega-3 fatty acid desaturase *fat-1*(*loss-of-function*; *lof*) mutant worm which is also sensitive to the oxidative stress agent paraquat. Triglyceride with three omega-3 PUFAs, trilinolenin [TG(54:9)], was sufficient to induce lipid peroxidation while deuterated TG(54:9) [D-TG(54:9)] was protective. D-TG(54:9) reduced the oxidative stress response in addition of significantly extending the lifespan of *fat-1*(*lof*) under normal and oxidative stress conditions. These findings demonstrate that D-TG(54:9) is adequate to prevent the propagation of ROS-induced molecular damages resulting in a significant extension of lifespan.

## Materials and Methods

### Statistics

Error bars indicate standard error of the mean (SEM), calculated from at least three biological replicates, unless otherwise indicated. *P*-values were calculated using one-way ANOVA with Tukey’s test or log-rank test for lifespan, unless otherwise indicated and reported as *P*-values. All statistical tests were performed using GraphPad Prism 7 software.

### *C. elegans* Strains, Bacterial Strains, and Food Additive

All strains were grown at 20°C using standard *C. elegans* methods as previously described (Koh et al., 2018). Nematode growth media (NGM) agar plates were seeded with *Escherichia coli* strain OP50 for normal growth. *C. elegans* strains wild type N2, *fat-1*(*bx24*), *mev-1*(*tk22*), *gst-4p::GFP::NLS*(*cl2166*), *sod-3p::GFP*(*cf1553*) and bacteria strains *OP50* were gifted from the *Caenorhaditis* Genetics Center. Trilinolenin [TG(54:9)] was obtained from Nu-Chek Prep and deuterated at bis-allylic position as previously described (Smarun et al., 2017) to obtained a mixture of 76% deuterated TG(54:9) [D-TG(54:9)]. Lipids were stored into an atmosphere of argon to prevent lipid oxidation. Lipids were freshly dissolved in PBS buffer (137 mM NaCl, 10 mM phosphate, 2.7 mM KCl, pH 7.4) containing 0.1% Triton X-100 or kept at -80°C for later use prior to supplementing NGM agar plate as previously described (Deline et al., 2013). Butylated hydroxytoluene (BHT) and paraquat (PQ) were obtained from Sigma and Acros Organics, respectively.

### Lifespan Assays

Lifespan assays were performed at 20°C as previously described (Apfeld and Kenyon, 1999). Synchronized animals were transferred to NGM plates containing 0.2 mM BHT, 1 mM paraquat (PQ), 0.48 mM TG(54:9), or 0.48 mM D-TG(54:9), when indicated. Pyrimidine analog 5-fluoro-2’-deoxyuridine (FUdR, Sigma; St. Louis) was added at 50 μM to pre-fertile young adult worms to prevent development of progeny. Adults were scored manually as dead or alive every 2–3 day. Nematodes which ceased pharyngeal pumping and had no respond to gentle stimulation were recorded as dead. Those worms that were male, crawled off the plate or non-natural death were censored.

### Lipid Analysis

Synchronized L1 animals were transferred to NGM plates containing 0.48 mM TG(54:9), 0.48 mM D-TG(54:9) or carrier. Approximately 10,000 L4 to young adult worms were harvested and washed thoroughly with M9 buffer and lyophilised overnight (Vertis, Warminster, PA, United States). FAs were esterified to fatty acid methyl esters (FAME) with 300 μl of 1.25 M HCl-methanol for 1 h at 80°C. FAMEs were extracted three times with 1 ml of hexane. Combined extracts were dried under nitrogen, resuspended in 100 μl hexane. FAMEs were separated by gas chromatography with flame ionization detector (GC-FID; GC-2014; Shimadzu, Kyoto, Japan) using an ULBON HR-SS-10 50 m × 0.25 mm column (Shinwa, Tokyo, Japan). Supelco 37 component FAME mix was used to identify corresponding FAs (Sigma-Aldrich, St. Louis, MO, United States). Data was normalized using internal standard pentadecanoic acid (C15:0) and worm dry weight.

### TBARS Assay

Synchronized L1 mev-1 mutants were transferred to NGM plates containing 0.1 mM BHT, 0.48 mM TG(54:9), 0.48 mM D-TG(54:9) or carrier for 54 h, after which they were transferred on supplemented plates with 8 mM paraquat when indicated for 24 h. Approximately 1,000 L4 to adult worms were harvested and washed thoroughly with M9 buffer, resuspended in 300 μl RIPA buffer (50 mM Tris–HCL pH7.5, 150 mM NaCl, 2 mM EDTA, 1% NP-40, 0.1% SDS), lysed with 1 mm silica beads by bead beating. Protein concentration was carried out using the Bicinchoninic Acid (BCA) Protein Assays kit following manufacturer’s protocol (Sigma; St. Louis). Assays for lipid peroxidation, using the thiobarbituric acid reactive substrate (TBARS) kit were performed following manufacturer’s protocol (Cayman Chemical).

### BODIPY-C11 Lipid Peroxidation Reporter Assay

Synchronized L1 mev-1 animals were transferred to NGM plates containing 0.1 mM BHT, 0.48 mM TG(54:9), 0.48 mM D-TG(54:9) or carrier and supplemented with 5 mM paraquat when indicated. L4 to young adult worms were transferred to 10 μM BODIPY 581/591 undecanoic acid (BODIPY^581-591^-C11) solution and stained for 30 min and washed three times with M9. Images were captured using confocal fluorescence microscope Zeiss LSM 710 microscope with a 20 × objective (Carl Zeiss MicroImaging). Oxidized and non-oxidized BODIPY^581-591^-C11 were excited at 488 and 568 and images were collected from emission at 530(30) and 590(30) nm, respectively. Fluorescence signal ratio of oxidized to non-oxidized BODIPY was normalized to carrier.

### Fluorescence Microscopy

Synchronized L1 *gst-4p::GFP* or *sod-3p::GFP* worms were transferred to NGM plates containing 0.48 mM TG(54:9), 0.48 mM D-TG(54:9) or carrier and supplemented with 5 mM paraquat when indicated. To quantify GFP signal, worms were immobilized with 25 mM tetramisole and mounted on 2% agarose pad. Images were captured using Zeiss Axiovert 200 M fluorescence microscope with a 20 × objective. Images were stitched, and total fluorescence were quantified using Fiji ImageJ software.

### Egg Counting Assay

Synchronized L1 WT and *fat-1*(*lof*) animals were transferred to NGM plates containing 0.48 mM TG(54:9), or 0.48 mM D-TG(54:9) and supplemented with 5 μM 5-fluoro-2’-deoxyuridine (FUdR, Sigma; St. Louis) to facilitate the counting of the eggs. The fertility of the worms was scored by counting eggs on day 5 and 6 after the transfer of the adults to a new plate.

## Results

### Worm Lacking *fat-1* as a Model to Mimic Human Dietary Needs of Omega-3

In human, FAs lineloic acid (LA; omega-6) and α-linolenic acid (ALA; omega-3) are essentials (MacLean et al., 2004). In contrast, *C. elegans* synthesize both LA and ALA consequently we selected omega-3 fatty acid desaturase *fat-1*(*loss-of-function*; *lof*) mutant animal to mimic the human dietary requirement of ALA (Watts and Browse, 2002; Figure 1A). We tested different ROS agents including paraquat (PQ) and 95% oxygen (data not shown). Paraquat is reduced into radical from the mitochondrial leaking electrons to induce the production of superoxide radicals such as superoxide anion radical (O_2_^∙-^). In turn, hydroperoxyl radicals HO_2_^∙^ modify PUFAs through oxidative damage in a chain-propagation fashion (Bielski et al., 1983; De Grey, 2002). As low levels of oxidative stress has been reported to increase the lifespan of *C. elegans* (Wei and Kenyon, 2016) while high levels decrease lifespan (Schaar et al., 2015), we carried out a lifespan assay with *fat-1*(*lof*) worms. As expected, high concentration of 1 mM paraquat significantly reduced the lifespan of *fat-1*(*lof*) worms compared to carrier (Figure 1B and Table 1). This result indicates that *fat-1*(*lof*) worms are sensitive to the production of ROS by the toxic level of paraquat. We also subjected *fat-1*(*lof*) worms to the antioxidant BHT but the lifespan was unchanged compared to carrier. This result suggests that *fat-1*(*lof*) basal level of ROS is not harmful and neutralising ROS further with antioxidant does not influence *fat-1*(*lof*) longevity.

**Table 1:** Lifespan analysis.

Strains	Treatment^∗^	Mean lifespan ± SEM (days)	75%	% change to control	Number of animals	*P*-values versus carrier	Figure
*fat-1*(*lof*)	carrier	14.8 ± 0.7	19		58/60		Figure 1C
	PQ	11.7 ± 0.6	13	-21	55/60	0.0002	
	BHT	15.1 ± 0.6	18	+3	51/60	0.9057	
*fat-1*(*lof*)	TG(54:9)	19.6 ± 0.6	21		59/60		Figure 4A
	D-TG(54:9)	21.4 ± 0.6	23	+9^∗∗^	56/60	0.0230^∗∗^	
*fat-1*(*lof*)	carrier + PQ	11.7 ± 0.6	13	0	55/60		Figure 4B
	TG(54:9) + PQ	13.3 ± 0.8	18	+14	55/60	0.5070	
	D-TG(54:9) + PQ	16.7 ± 1.0	18	+43	42/60	0.0001	
*fat-1*(*lof*)	carrier	25.6 ± 0.8	29		58/60		biological replicates for Figure 1C
	PQ	22.9 ± 0.9	25	-10	48/60	0.0725	
	BHT	23.7 ± 0.9	27	-8	50/60	0.1952	
*fat-1*(*lof*)	TG(54:9)	12.5 ± 0.6	16		42/60		biological replicates for Figure 4A
	D-TG(54:9)	16.4 ± 0.7	18	+31^∗∗^	47/60	<0.0001^∗∗^	

*^∗^BHT, 0.2 mM butylated hydroxytoluene; PQ, 1 mM paraquat; TG(54:9), 0.48 mM trilinolenin; D-TG(54:9), 0.48 mM deuterated trilinolenin. ^∗∗^compared to TG(54:9) treatment.*

### Trilinolenin Aggravates *fat-1(lof)* Infertility Phenotype

The protective effect of D-PUFAs against ROS has been demonstrated in yeast (Hill et al., 2011; Cotticelli et al., 2013; Andreyev et al., 2015), in primary co-cultures of neurons and astrocytes (Angelova et al., 2015) as well as in AD mouse model *Aldh2^-/-^* (Elharram et al., 2017) by supplementing the media with deuterated LA or ALA (Figure 1C; Hill et al., 2011; Cotticelli et al., 2013; Andreyev et al., 2015). We generated deuterated ALA from trilinolenin [TG(54:9)] to obtain deuterated trilinolenin [D-TG(54:9)] as triglycerides reflect human intake mainly consisting of esterified FAs (Figure 1D). The replacement of hydrogen by deuterium at the potential four bis-allylic CH_2_ group between the two double bonds was effective at 97.5% (data not shown) using our recently reported site-specific deuteration of polyunsaturated alkenes method (Smarun et al., 2017). To ensure that *C. elegans* ingests and metabolizes D-TG(54:9) as well as non-deuterated TG(54:9), we fed L1 larvae *fat-1*(*lof*) mutant with standard diet on NGM plates supplemented with 0.48 mM TG(54:9), D-TG(54:9), or the carrier (0.1% Triton X-100 in PBS). *C. elegans* is typically fed *E. coli* OP50 bacteria in which PUFAs are naturally absent but can be easily incorporated if supplemented to the media (Webster et al., 2013). Worms were harvested at stage larva 4 (L4)/young adult and lyophilized. Dried worms were subjected to derivatisation with hydrogen chloride in methanol to generate FAME. FAMEs were extracted with hexane and separated on GC-FID using a capillary column (Ulbon HR-SS-10). We observed a significant accumulation of eicosatetraenoic acid (ETA) and eicosapentaenoic acid (EPA) in *fat-1*(*lof*) fed D-TG(54:9) while the other changes were statistically non-significant compared to carrier (Figure 1E). This result indicates that deuterated α-linolenic acid is metabolized by *C. elegans* as a precursor to synthesize PUFAs with higher degrees of unsaturation.

Next, we asked if D-TG(54:9) is toxic to *fat-1*(*lof*) mutant by measuring its fertility which is diminished by ROS (Alcantar-Fernandez et al., 2018). As Δ9 fatty acid desaturases *fat-6*;*fat-7* double mutant exhibits lower fertility (Brock et al., 2007), we monitored the number of laid eggs in wild-type (WT, N2) and *fat-1*(*lof*) mutant with standard diet on NGM plates supplemented with 0.48 mM TG(54:9), D-TG(54:9), or the carrier. On carrier, *fat-1*(*lof*) mutant laid a significant lower amount of eggs compared to WT (Figure 1F). Similarly, *fat-1*(*lof*) mutant, fed TG(54:9), laid a similar amount of eggs per worm compared to *fat-1*(*lof*) mutant on carrier. To our surprise, *fat-1*(*lof*) mutant, fed D-TG(54:9), laid a similar amount of eggs per worm compared to WT. This result suggests that D-TG(54:9) promotes fertility in *fat-1*(*lof*) mutant while TG(54:9) might promote lipid peroxidation.

### Trilinolenin Is Sufficient to Induce Lipid Peroxidation While Deuterated Trilinolenin Is Protective Upon Oxidative Stress

**FIGURE 2:**
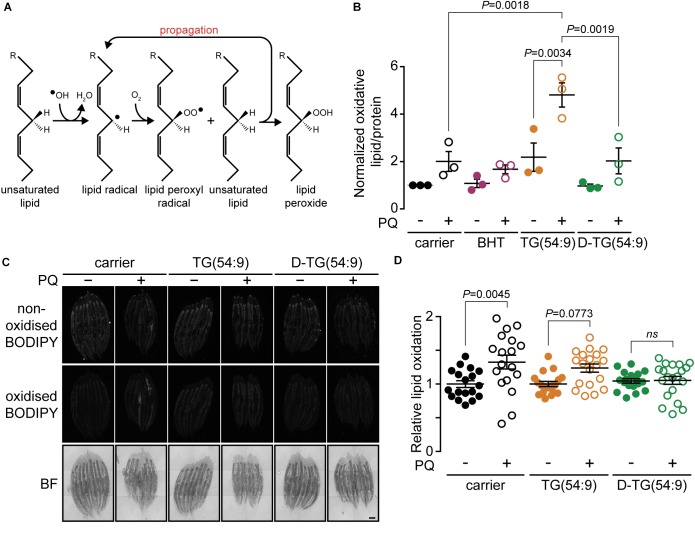
Trilinolenin is sufficient to induce lipid peroxidation while deuterated trilinolenin is protective. **(A)** Schematic representation of lipid peroxidation. **(B)** Normalized oxidized lipids of *mev-1*(*lof*) worms fed OP50 supplemented with carrier, 0.1 mM BHT, 0.48 mM trilinolenin [TG(54:9)] or 0.48 mM deuterated trilinolenin [D-TG(54:9)] and subjected to 8 mM paraquat (PQ) when indicated. **(C)** Representative confocal fluorescence images of *mev-1*(*lof*) worms fed OP50 supplemented with carrier, 0.48 mM trilinolenin [TG(54:9)] or 0.48 mM deuterated trilinolenin [D-TG(54:9)] and subjected to 5 mM paraquat (PQ) when indicated. Scale bar, 100 μm. **(D)** Quantification of oxidized to non-oxidized BODIPY581/591-C11 from **(C)**.

As PUFAs contribute to the generation of oxidative stress through the propagation of lipid peroxide (Porter et al., 1995; Yin et al., 2011; Figure 2A), we hypothesized that TG(54:9) will intensify paraquat-induced lipid peroxidation. Lipid peroxidation was monitored by measuring thiobarbituric acid reactive substances (TBARS) (Lagman et al., 2015) in lipid peroxidation-sensitive *mev-1*(*lof*) mutant worms (Labuschagne et al., 2013). The protein MEV-1 is the homolog of succinate dehydrogenase cytochrome b560 subunit of the mitochondrial respiratory chain complex II (Ishii et al., 1998). Synchronized L1 *mev-1*(*lof*) mutants were grown on NGM plates seeded with bacteria supplemented with carrier, BHT, TG(54:9), or D-TG(54:9). Subsequently, L4/young adult *mev-1*(*lof*) mutants were exposed to 24 h of paraquat. Paraquat alone was insufficient to significantly increase the accumulation of lipid peroxide compared to carrier alone (Figure 2B). On the other hand, *mev-1*(*lof*) mutants grown on TG(54:9) and subjected to paraquat exhibited a dramatic increase in lipid peroxides compared to paraquat alone and to TG(54:9) in the absence of paraquat. This result reinforces the role of PUFAs in catalyzing the propagation of ROS through lipid peroxidation (Yang et al., 2016). D-TG(54:9) was sufficient to prevent the propagation of paraquat-induced ROS through lipid peroxidation. This result indicates that D-PUFAs, in the form of triacylglycerol, are resistant to ROS and thus preventing further cellular damages.

To further assess the role of D-TG(54:9) in preventing the formation of lipid peroxides, we monitored *in vivo* fluorescence of the sensor BODIPY^581/591^ undecanoic acid (BODIPY^581/591^-C11). The sensor emission peak shifts from 590 to 510 nm when the polyunsaturated butadienyl domain is oxidized (Naguib, 1998; Aldini et al., 2001). *mev-1*(*lof*) mutants were grown and treated as for the TBARS assay. In contrast to the TBARS assay, paraquat alone was sufficient to significantly induce lipid peroxidation (Figure 2C,D). This suggests that BODIPY^581/591^-C11 is more sensitive than the TBARS assay to monitor lipid peroxidation in *C. elegans* as previously reported in different cell types (Dominguez-Rebolledo et al., 2010). In contrast to the TBARS assay, TG(54:9) combined with paraquat did not induce lipid peroxidation further compared to paraquat alone in *mev-1*(*lof*) mutants. D-TG(54:9) was sufficient to protect *mev-1*(*lof*) mutants against paraquat-induced lipid peroxidation. As the BODIPY-C11 lipid peroxidation reporter assay yielded small differences, BODIPY possibly failed to be incorporated evenly through the animal and perhaps not sufficiently where most of lipid peroxidation occurs. Together with the TBARS assay, these results demonstrate that D-PUFA is sufficient to prevent lipid peroxidation in *C. elegans*.

### Deuterated Trilinolenin Reduces Oxidative Stress Response

**FIGURE 3:**
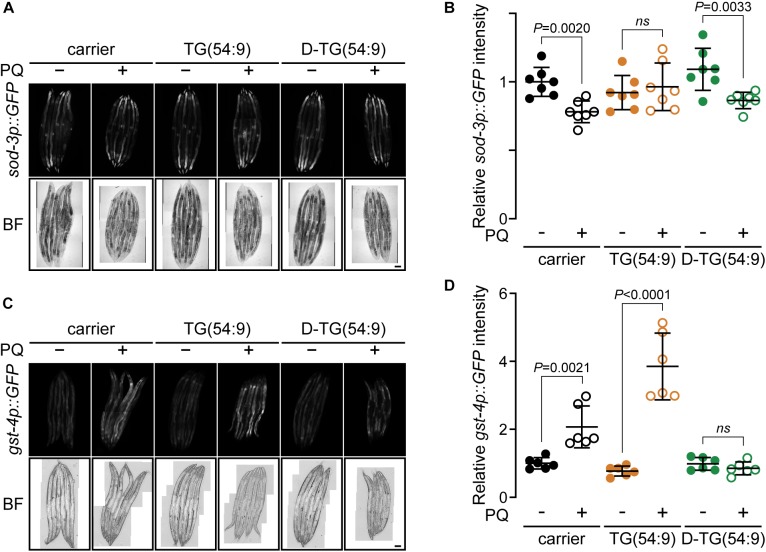
Deuterated trilinolenin reduces oxidative stress response. **(A)** Representative confocal fluorescence images of *sod-3p::GFP* transgenic worms fed OP50 supplemented with carrier, trilinolenin [TG(54:9)] or deuterated trilinolenin [D-TG(54:9)] and subjected to 5 mM paraquat (PQ) when indicated. Scale bar, 100 μm. **(B)** Quantification of A. **(C)** Representative confocal fluorescence images of *gst-4p::GFP::NLS* transgenic worms treated as in A fed OP50 supplemented with carrier, trilinolenin [TG(54:9)] or deuterated trilinolenin [D-TG(54:9)] and subjected to 5 mM paraquat (PQ) when indicated. Scale bar, 100 μm. **(D)** Quantification of C.

To further assess the role of D-TG(54:9) in preventing the propagation of lipid peroxide, we monitored the oxidative stress response *in vivo*. We monitored the expression of the reporter protein GFP under the promotor *sod-3* (*sod-3p::GFP*) (Xu and Kim, 2012). SOD-3 is a mitochondrial superoxide dismutase which has been reported to increase transcriptionally as a result of oxidative stress (An and Blackwell, 2003). These worms were grown and treated as per the TBARS assay. No significant increase in the expression of *sod-3p::GFP* was observed across the different conditions (Figure 3A,B). As this assay was inconclusive, we used the transgenic worm expressing GFP tagged with a nuclear localisation signal under the promotor *gst-4* (*gst-4p::GFP::NLS*) (Paek et al., 2012). GST-4 is a glutathione S-transferase protein that responds to oxidative stress by an increase at the transcription and translational levels (Link and Johnson, 2002). Glutathione S-transferases protect cells against lipid peroxidation (Yang et al., 2001). *gst-4p::GFP::NLS* worms were grown with triacylglycerol and paraquat as per the TBARS assay. The expression of *gst-4p::GFP::NLS* was significantly increased in the presence of paraquat and further increase with the addition of TG(54:9) (Figures 3C,D). On the other hand, no significant change in GFP expression was observed in animals fed D-TG(54:9) in the absence or the presence of paraquat. Consistent with the TBARS assay, these findings further demonstrate that TG(54:9) is harmful by exponentially propagating paraquat-induced ROS while D-TG(54:9) exhibit a strong protective effect.

### Deuterated Trilinolenin Extend Lifespan Under Both Normal and Oxidative Stress Conditions

**FIGURE 4:**
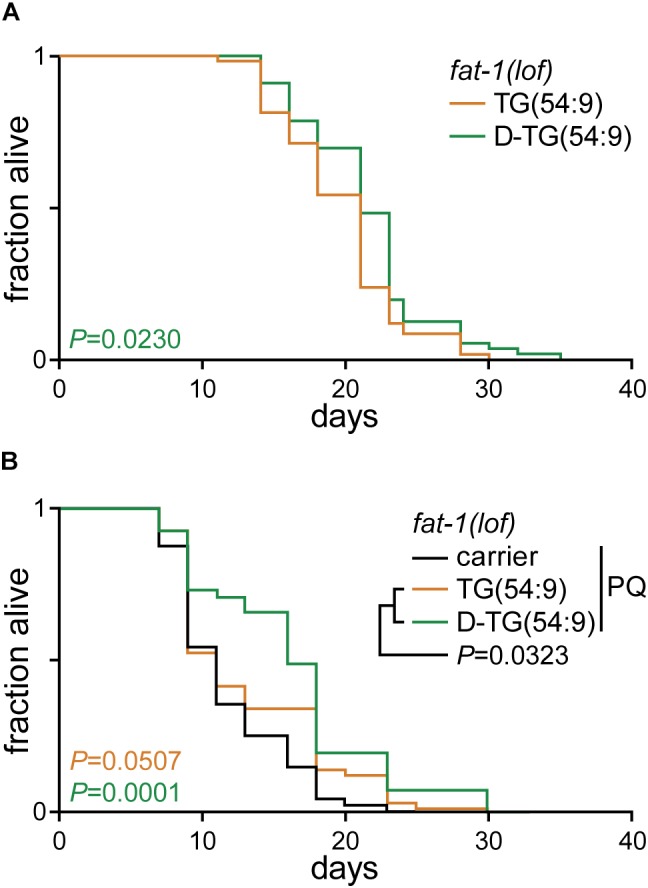
Deuterated trilinolenin reduces lipid peroxidation. **(A)** Lifespan assay of *fat-1*(*lof*) worms fed OP50 supplemented with 0.48 mM trilinolenin [TG(54:9)] or 0.48 mM deuterated trilinolenin [D-TG(54:9)]. Log-rank test to control. **(B)** Lifespan assay of worms fed OP50 supplemented with carrier, trilinolenin [TG(54:9)] or deuterated trilinolenin [D-TG(54:9)] and subjected to 1 mM paraquat (PQ). Log-rank test to control.

As D-TG(54:9) reduces oxidative stress, we carried out a lifespan assay of *fat-1*(*lof*) worms fed normal diet supplemented with either TG(54:9) or D-TG(54:9). D-TG(54:9) significantly extended the lifespan of worms compared to the supplementation with TG(54:9) (Figure 4A and Table 1). This suggests that D-TG(54:9) might be sufficient to prevent endogenous ROS-induced cellular damages associated with aging. To further assess the role of D-TG(54:9) on the lifespan of *fat-1*(*lof*) worms, we exposed the animals to paraquat from L1 stage. As expected, the lifespan of *fat-1*(*lof*) worms exposed to paraquat was not significantly extended by TG(54:9) (Figure 4B and Table 1). As *fat-1*(*lof*) worms are unable to synthesize several PUFAs (Figure 1A), TG(54:9) might be necessary as a precursor of PUFAs ETA and EPA to extend the lifespan although the animals are under oxidative stress condition. It should be noted that worms were exposed to a fifth of the paraquat concentration used to monitor lipid peroxidation and oxidative stress. The lifespan of *fat-1*(*lof*) worms exposed to paraquat was further extended with D-TG(54:9) compared to paraquat alone and to TG(54:9) in combination with paraquat. This result indicates that D-TG(54:9) is sufficient to promote longevity. Potentially, supplementing a human diet with D-PUFAs might be sufficient to decelerate aging and to prevent the progression of age-associated diseases.

## Discussion

Chemically reinforced essential FAs have the potential to target age-related diseases such as Alzheimer’s, Friedreich’s ataxia and other neurological conditions. Substituting the atoms of hydrogen at the bis-allylic methylene of essential polyunsaturated FAs with isotope deuterium prevents substantial chemical damage. Recently, we developed a novel synthesis approach to reinforced natural FAs (Smarun et al., 2017), which could potentially be scaled up to industrial quantities. These deuterated FAs have the advantage to prevent harmful accumulation of ROS by disfavouring the formation of lipid peroxides while antioxidants are poorly transported within the cell. The results presented in this study show that D-TG(54:9) is sufficient to prevent the accumulation of lipid peroxides in animals and to reduce the accumulation of ROS. We used four different approaches which, together, clearly demonstrate the protective effect of deuterated trilinolenin against paraquat-induced oxidative stress while non-deuterated trilinolenin promotes the propagation of lipid peroxide. Supplementing the diet with D-PUFAs was anticipated to delay aging (Shchepinov, 2007). As predicted, D-TG(54:9) significantly extends the lifespan of worms under both normal and oxidative stress conditions when compared to non-deuterated trilinolenin [TG(54:9)]. It should be noted that D-PUFA must be in low abundance to be beneficial (Kinghorn et al., 2015). Taken together, we have developed a toolkit to monitor the protective role of deuterated FAs against age-related model diseases as well as longevity, allowing future high-throughput discoveries.

During the late nineties, a large boom of research on dietary antioxidants generated hopes as potential agents to slow aging and to prevent the development of ROS-related diseases. However, mixed results emerged from studies conducted in humans (Moller and Loft, 2002). It became clear that human cells generate a certain number of free radicals that play an important role in cellular functions (Halliwell, 2011; Sena and Chandel, 2012; Yan, 2014). A balance of antioxidants and reactive species must be kept *in vivo*, and supplementation of dietary antioxidants might compromise this delicate equilibrium. More importantly, as PUFAs belong to the group of essential nutrients that must be supplied with diet, it was proposed that PUFAs with deuterated bis-allylic methylenes could represent a novel approach to fight against ROS-initiated attacks, leading to lessening the influence of aging and age-associated diseases on human life.

The protective effect of D-PUFAs against ROS has been demonstrated in yeast and in primary co-cultures of neurons and astrocytes (Hill et al., 2011; Cotticelli et al., 2013; Andreyev et al., 2015; Angelova et al., 2015). As unicellular organisms uniformly absorb FAs, it is imperative to assess the protective role of D-PUFA that is absorbed in the intestine and disseminated to different tissues of a multicellular organism. Recently, D-PUFAs was shown to improve cognition and to reduce lipid peroxidation in the brain of several neurodegenerative disease models (Elharram et al., 2017; Hatami et al., 2018; Raefsky et al., 2018). Similarly in *C. elegans*, we have demonstrated that H-PUFA promotes, while D-PUFA reduces, both lipid peroxidation and oxidative stress during paraquat-induced oxidative stress. For the first time, we have demonstrated the beneficial effect of D-PUFAs in extending longevity. In future, *C. elegans* could be used to validate the beneficial role of D-PUFAs in modulating the lifespan of different disease models.

## Data Availability

All datasets generated for this study are included in the manuscript and/or the supplementary files.

## Author Contributions

GT conceived the study, wrote the original draft of the manuscript, and acquired the funding. CB-C and GT performed the methodology. CB-C and LW contributed to formal analysis. CB-C investigated the study. CB-C, AS, MS, and DV performed the resources. MS and GT wrote, reviewed, and edited the manuscript.

## Conflict of Interest Statement

MS owns stocks in Retrotope. The remaining authors declare that the research was conducted in the absence of any commercial or financial relationships that could be construed as a potential conflict of interest.
